# Supplementary material for: Investigating the risk factors for seroprevalence and the correlation between CD4+ T-cell count and humoral antibody responses to Toxoplasma gondii infection amongst HIV patients in the Bamenda Health District, Cameroon
Source: PLoS One. 2021 Dec 2;16(12):e0256947. doi: 10.1371/journal.pone.0256947 (PMC8638881; doi:10.1371/journal.pone.0256947)
Supplement: Attachment — Submitted filename: PONE-D-20-04369_R1_reviewer.pdf [file pone.0256947.s003.pdf]

PLOS ONE

Investigating the risk factors for seroprevalence and the correlation between CD4+ T-cell count and humoral antibody responses to Toxoplasma gondii infection amongst HIV patients in the Bamenda Health District, Cameroon.

--Manuscript Draft--

**Manuscript Number:**	PONE-D-20-04369R1
**Article Type:**	Research Article
**Full Title:**	Investigating the risk factors for seroprevalence and the correlation between CD4+ T-cell count and humoral antibody responses to Toxoplasma gondii infection amongst HIV patients in the Bamenda Health District, Cameroon.
**Short Title:**	Risk factors for sero-prevalence of Toxoplasmosis and role of CD4+ as determinant of acute infection in HIV
**Corresponding Author:**	Fang Eugene Enah, M.Sc<br>University of Buea<br>Buea/Cameroon, CAMEROON
**Keywords:**	T. gondii, sero-prevalence, Bamenda Health District, CD4+ T-cells, HIV.
**Abstract:**	Background : Toxoplasmosis is caused by an obligate intracellular tissue protozoan parasite, Toxoplasma gondii that infect humans and other warm-blooded animals. Transmission to humans is by eating raw or inadequately cooked infected meat or through ingestion of oocysts that cats have passed in faeces. Studies have shown life-threatening and substantial neurologic damage in immunocompromised patients; however, 80% of humans remain asymptomatic. The aim of this study was to determine the seroprevalence of T. gondii infection in HIV positive patients and the risk factors associated with the infection, and to investigate the threshold of CD4+ T-cell count associated with acute T. gondii infection in the Bamenda Health District of the North West Region of Cameroon.<br>Methods : A cross-sectional study was conducted, in which 325 HIV patients were recruited for administration of questionnaire, serological diagnosis of T. gondii and measurement of CD4+ T-cell count. Bivariate and multivariate logistic regression was used to identify risk factors associated with T. gondii infection while the linear regression was used to investigate the relationship between CD4+ T-cell count and antibody levels against T. gondii .<br>Results : The findings showed that, majority (45.8%) of HIV patients suffered from chronic (IgG antibody) infection, and 6.5% from acute (IgM and IgM/IgG antibody) toxoplasma infection. The overall sero-prevalence of T. gondii infection amongst HIV patients was 52.3%. On the whole, 43 men (45.7%) and 127 women (55%) presented with anti- T. gondii antibodies; however, there was no significant difference amongst males and females who were positive to T. gondii infection (p =0.131). Marital status (p =0.0003), contact with garden soil (p =0.0062), and garden ownership (p =0.009), were factors that showed significant association with T. gondii infection. There was no significant difference (p = 0.909) between the mean CD4+ T-cell count of HIV patients negative for toxoplasma infection (502.7 cells/mL), chronically infected with T. gondii (517.7 cells/mL) and acutely infected with T. gondii (513.1 cells/mL). CD4+ T-cell count was neither a predictor of IgM antibody titer (r =0.193, p =0.401), nor IgG antibody titer (r = 0.149, p =0.519) amongst HIV patients acutely infected with T. gondii .<br>Conclusion: The findings from this study underscore the need to implement preventive and control measures to fight against T. gondii infection amongst HIV patients in the Bamenda Health District.
**Order of Authors:**	Fang Eugene Enah, M.Sc
	Nyasa Raymond Babila
	Ndi Emmanuel Menang
	Denis Zofou
	Tebit Emmanuel Kwenti

Powered by Editorial Manager® and ProduXion Manager® from Aries Systems Corporation

	Pafoule Edith Lepezeu
	Vincent Titanji
	Roland N. Ndip
**Response to Reviewers:**	Response to Reviewers.<br>Dear reviewers kindly fine in this later the response to the questions posted.<br>The topic of the manuscripts has been modified to “Investigating the risk factors for seroprevalence and the correlation between CD4+ T-cell count and humoral antibody responses to Toxoplasma gondii infection amongst HIV patients in the Bamenda Health District, Cameroon” to tie the research with the scientific outcome. the map have been removed because it does not significantly add value to the article.<br>Information about the pretest procedure of the question is as described in line 124 in the questionnaire administration sub heading.<br>During analysis, the numeric variables followed an abnormal distribution and the analysis of variance test was used.<br>Determining the relationship between CD4+ T-cell count to antibody titer was to evaluate the effect of the parasite on the immune system in the study population. The investigator captured the aspect of risk factors to ascertain the epidemiology situation of the parasite in the study population. The investigator used a highly specific and sensitive screening test to ascertain the status of infection of the parasite among the study population. It is also true that, the presence of IgM antibodies is the indication of onset of infection and/or recontamination. The inclusion criteria have been addressed in the revised manuscript line 119 indicating the patients were for their routine drug pick up.<br>The analysis of CD4+T-cell counts among toxoplasma negative and positive patients was to proof the hypothesis that say immune depression (Low CD4+ T-cell count) futher puts the patients in danger to Toxoplasmosis complications.
**Additional Information:**	
**Question**	**Response**
**Financial Disclosure**<br><br>Enter a financial disclosure statement that describes the sources of funding for the work included in this submission. Review the submission guidelines for detailed requirements. View published research articles from *PLOS ONE* for specific examples.<br><br>This statement is required for submission and **will appear in the published article** if the submission is accepted. Please make sure it is accurate.	The author(s) received no specific funding for this work.

Powered by Editorial Manager® and ProduXion Manager® from Aries Systems Corporation

**Unfunded studies**<br>Enter: *The author(s) received no specific funding for this work.*<br><br>**Funded studies**<br>Enter a statement with the following details:<br>• Initials of the authors who received each award<br>• Grant numbers awarded to each author<br>• The full name of each funder<br>• URL of each funder website<br>• Did the sponsors or funders play any role in the study design, data collection and analysis, decision to publish, or preparation of the manuscript?<br>• **NO** - Include this sentence at the end of your statement: *The funders had no role in study design, data collection and analysis, decision to publish, or preparation of the manuscript.*<br>• **YES** - Specify the role(s) played.<br><br>* typeset	
**Competing Interests**<br><br>Use the instructions below to enter a competing interest statement for this submission. On behalf of all authors, disclose any competing interests that could be perceived to bias this work—acknowledging all financial support and any other relevant financial or non-financial competing interests.<br><br>This statement **will appear in the published article** if the submission is accepted. Please make sure it is accurate. View published research articles from *PLOS ONE* for specific examples.	The authors have declared that no competing interests exist.

Powered by Editorial Manager® and ProduXion Manager® from Aries Systems Corporation

**NO authors have competing interests**<br><br>Enter: *The authors have declared that no competing interests exist.*<br><br>**Authors with competing interests**<br><br>Enter competing interest details beginning with this statement:<br><br>*I have read the journal's policy and the authors of this manuscript have the following competing interests: [insert competing interests here]*<br><br>* typeset	
**Ethics Statement**<br><br>Enter an ethics statement for this submission. This statement is required if the study involved:<br><br>• Human participants<br>• Human specimens or tissue<br>• Vertebrate animals or cephalopods<br>• Vertebrate embryos or tissues<br>• Field research<br><br>Write "N/A" if the submission does not require an ethics statement.<br><br>General guidance is provided below. Consult the submission guidelines for detailed instructions. **Make sure that all information entered here is included in the Methods section of the manuscript.**	Ethical clearance for this study was obtained from the University of Buea, Faculty of Health Science (FHS) Institutional Review Board (IRB), reference number 2018/2244UB/SG/IRB/FHS and the Bamenda Regional Hospital Institutional Review Board, reference number 26/APP/RDPH/RHB/IRB. Administrative authorization was obtained from the Regional Delegation of Public Health for the North West Region (RDPH), reference number 233/ATT/NWR/RDPH. Only consenting participants from the study population were recruited into the study, and they all signed the informed consent form. For participants below the age of 21 years old, a signed ascent form was also obtained from their parents or guardians to enable them take part in the research.

Powered by Editorial Manager® and ProduXion Manager® from Aries Systems Corporation

**Format for specific study types**<br><br>**Human Subject Research (involving human participants and/or tissue)**<br>• Give the name of the institutional review board or ethics committee that approved the study<br>• Include the approval number and/or a statement indicating approval of this research<br>• Indicate the form of consent obtained (written/oral) or the reason that consent was not obtained (e.g. the data were analyzed anonymously)<br><br>**Animal Research (involving vertebrate animals, embryos or tissues)**<br>• Provide the name of the Institutional Animal Care and Use Committee (IACUC) or other relevant ethics board that reviewed the study protocol, and indicate whether they approved this research or granted a formal waiver of ethical approval<br>• Include an approval number if one was obtained<br>• If the study involved *non-human primates*, add *additional details* about animal welfare and steps taken to ameliorate suffering<br>• If anesthesia, euthanasia, or any kind of animal sacrifice is part of the study, include briefly which substances and/or methods were applied<br><br>**Field Research**<br><br>Include the following details if this study involves the collection of plant, animal, or other materials from a natural setting:<br>• Field permit number<br>• Name of the institution or relevant body that granted permission	
**Data Availability**<br><br>Authors are required to make all data underlying the findings described fully available, without restriction, and from the time of publication. PLOS allows rare exceptions to address legal and ethical concerns. See the PLOS Data Policy and FAQ for detailed information.	Yes - all data are fully available without restriction

A Data Availability Statement describing where the data can be found is required at submission. Your answers to this question constitute the Data Availability Statement and **will be published in the article**, if accepted.<br><br>**Important:** Stating 'data available on request from the author' is not sufficient. If your data are only available upon request, select 'No' for the first question and explain your exceptional situation in the text box.<br><br>Do the authors confirm that all data underlying the findings described in their manuscript are fully available without restriction?	
**Describe where the data may be found in full sentences. If you are copying our sample text, replace any instances of XXX with the appropriate details.**<br><br>• If the data are **held or will be held in a public repository**, include URLs, accession numbers or DOIs. If this information will only be available after acceptance, indicate this by ticking the box below. For example: *All XXX files are available from the XXX database (accession number(s) XXX, XXX.)*.<br>• If the data are all contained **within the manuscript and/or Supporting Information files**, enter the following: *All relevant data are within the manuscript and its Supporting Information files.*<br>• If neither of these applies but you are able to provide **details of access elsewhere**, with or without limitations, please do so. For example:<br><br>*Data cannot be shared publicly because of [XXX]. Data are available from the XXX Institutional Data Access / Ethics Committee (contact via XXX) for researchers who meet the criteria for access to confidential data.*<br><br>*The data underlying the results presented in the study are available from (include the name of the third party*	All relevant data are within the manuscript and its Supporting Information files.

and contact information or URL).<br>• This text is appropriate if the data are owned by a third party and authors do not have permission to share the data.<br><br>* typeset	
Additional data availability information:	

Manuscript
Click here to access/download;Manuscript;Revised Manuscript.docx

Investigating the risk factors for seroprevalence and the correlation between CD4+ T-cell count and humoral antibody responses to *Toxoplasma gondii* infection amongst HIV patients in the Bamenda Health District, Cameroon.

Eugene Enah Fang[1], Raymond Babila Nyasa [1,2]*, Emmanuel Menang Ndi[2], Denis Zofou[2], Tebit Emmanuel Kwenti[3], Edith Pafoule Lepezeu [4], Vincent P. K. Titanji[2,5] and Roland N. Ndip[1,6]

1. Eugene Enah Fang, M.Sc student in the department of Microbiology and Parasitology, University of Buea, P. O. Box 63, Buea, Cameroon. Email: enah.fang@gmail.com.
2. Raymond Babila Nyasa; Department of Microbiology and Parasitology, University of Buea, P. O. Box 63, Buea, Cameroon. email: nyasab@yahoo.co.uk, Emmanuel Menang Ndi. Biotechnology Unit, Faculty of Science, University of Buea, P.O. Box 63, Buea, South West Region, Cameroon. Email: ndifoinsahlawrence@gmail.com and Denis Zofou. Biotechnology Unit, Faculty of Science, University of Buea, P.O. Box 63, Buea, South West Region, Cameroon. Email: zofden@yahoo.com.
3. Tebit Emmanuel Kwenti. Department of Medical Laboratory Sciences, Faculty of Health Science, University of Buea, P.O. Box 63, Buea, South West Region, Cameroon. Email: kwentitebit@yahoo.com.
4. Pafoule Lepezeu Edith. Bamenda Regional Hospital, P.O. Box 518, Bamenda, North West Region, Cameroon. Email: edithpafoule@gmail.com.
5. Vincent P. K. Titanji. Cameroon Christian University, P.O. Box 5, Bali, North West Region, Cameroon. Email: vpk.titanji@yahoo.com.
6 Roland N. Ndip. Laboratory for Emerging Infectious Diseases, University of Buea, P.O.Box 63, Buea, South West Region, Cameroon. Email: ndip3@yahoo.com; ndip.roland@ubuea.cm.

***Corresponding author:** Raymond Babila Nyasa, Department of Microbiology and Parasitology, University of Buea, P. O. Box 63, Buea, South West Region, Cameroon.
Email: nyasab@yahoo.co.uk , Tel. Number: (+237) 677427747

Abstract

Background: Toxoplasmosis is caused by an obligate intracellular tissue protozoan parasite, *Toxoplasma gondii* that infect humans and other warm-blooded animals. Transmission to humans is by eating raw or inadequately cooked infected meat or through ingestion of oocysts that cats have passed in faeces. Studies have shown life-threatening and substantial neurologic damage in immunocompromised patients; however, 80% of humans remain asymptomatic. The aim of this study was to determine the seroprevalence of *Toxoplasma gondii* infection in HIV positive patients and the risk factors associated with the infection, and to investigate the correlation between CD4+ T-cell count and toxoplasma specific antibodies as possible predictors of each other amongst HIV patients in the Bamenda Health District of the North West Region of Cameroon.

Methods: A cross-sectional study was conducted, in which 325 HIV patients were recruited for administration of questionnaire, serological diagnosis of *T. gondii* and measurement of CD4+ T-cell count. Bivariate and multivariate logistic regression was used to identify risk factors associated with *T. gondii* infection while the linear regression was used to investigate the relationship between CD4+ T-cell count and antibody levels against *T. gondii*.

Results: The findings showed that, majority (45.8%) of HIV patients suffered from chronic (IgG antibody) infection, and 6.5% from acute (IgM and IgM/IgG antibody) toxoplasma infection. The overall sero-prevalence of *T. gondii* infection amongst HIV patients was 50.5%. On the whole, 43 men (45.7%) and 127 women (55%) presented with anti- *T. gondii* antibodies; however, there was

no significant difference amongst males and females who were positive to *T. gondii* infection (*p*=0.131). Marital status (*p*=0.0003), contact with garden soil (*p*=0.0062), and garden ownership (*p*=0.009), were factors that showed significant association with *T. gondii* infection. There was no significant difference (*p* = 0.909) between the mean CD4+ T-cell count of HIV patients negative for toxoplasma infection (502.7 cells/mL), chronically infected with *T. gondii* (517.7 cells/mL) and acutely infected with *T. gondii* (513.1 cells/mL). CD4+ T-cell count was neither a predictor of IgM antibody titer (*r*=0.193, *p*=0.401), nor IgG antibody titer (*r*= 0.149, *p*=0.519) amongst HIV patients acutely infected with *T. gondii*.

Conclusion: The findings from this study underscore the need to implement preventive and control measures to fight against *T. gondii* infection amongst HIV patients in the Bamenda Health District.

Key words: *T. gondii*, sero-prevalence, Bamenda Health District, CD4+ T-cells, HIV.

Introduction.

Toxoplasmosis caused by the obligate intracellular parasite, *Toxoplasma. gondii (T. gondii)* is a globally prevalent zoonotic disease of warm-blooded animals, including man. Within the human population, the infection is clinically silent in immunocompetent host and accounts for focal encephalitis, headache, confusion, motor weakness and fever amongst patients with acquired immunodeficiency syndrome (AIDS) or immuno-compromised patients [1]. In the absence of treatment, the disease progression results in seizures, stupor, coma and finally death [2]. The infection is contracted through consumption of oocyst in contaminated water, food or from inadequately cooked infected meat. Other means of infection include transfusion and transplantation [3].

It would be useful to present data regarding the seroprevalence and risk factors associated with the infection amongst human immunodeficiency virus (HIV) of *T. gondii* from different areas of the world. Studies elsewhere have implicated consumption of raw garden produce, level of education and age as risk factors of the infection [4,5,6].

Furthermore, toxoplasmosis has been described as the commonest cerebral opportunistic infection in HIV infected patients [7] and a common cause of mortality amongst severely immune depressed HIV patients [8]. It is known that a CD4+ T-cell count of ≤200cells/mm^3 accounts for reactivation of latent infection in HIV patients [9]. It is therefore rational to think that the magnitude of humoral immune response (IgG and IgM) is a function of CD4+ T cell count, since following *T. gondii* infection, a rise of specific acquired immunity, including humoral immunity accompanied by parasite clearance from the body [10]. If this is true, then high antibody titer in HIV infected toxoplasma patients should positively correlate with CD4+ T cell count, and consequently, either may serve as a marker of the other

Methods.

Study area and setting

The study was carried out in the Bamenda Health District (BHD), located on the High Western Plateau of Cameroon from August to October 2018. Bamenda is an urban setting, which is both the Administrative Headquarter of Mezam Division and the North West Region of Cameroon. The weather is warm and wet in the rainy season, which last from March to October. In the dry season (November to February), the mornings are generally very cold, and the afternoons are very hot compared to the rainy season. This cosmopolitan city is made up of mainly civil servants and business men and a majority of the population is partly involved in small scale farming. The BHD has a population of over 350,000 inhabitants. It comprises of 17 health areas, 14 public, and 4 faith-based health facilities (1 Baptist, 1 Presbyterian and 2 Catholics) with the Bamenda regional

hospital (public) being the leading health facility. Amongst the 18 health facilities, there are only four HIV treatment centers (Bamenda regional hospital, Nkwen sub divisional medicalised health center, Nkwen Baptist hospital and Mezam polyclinic) in BHD.

Study design

A cross sectional study was conducted in the Bamenda health district where data was collected from August to October 2018. Participants were recruited by a convenient sampling technique at the HIV treatment centers of the Bamenda regional hospital and the Nkwen sub divisional medicalised health center. The Bamenda Regional Hospital being the referral hospital and leading in terms of the HIV/AIDS patient population in the health district was purposively chosen for this study, while the Nkwen sub divisional medicalized health center was randomly selected from the other three HIV treatment centers within the health district. Assuming a prevalence of 69.6% from a previous study elsewhere (Yaounde) in Cameroon [11], the sample size was calculated using the formula of Suresh and Chandrashekara [12]. A value of 323 was achieved which was rounded up to 325 during implementation. Three hundred and twenty-five consenting HIV patients 12 years old and above, who were registered at the treatment centers as residents in the BHD were enrolled into the study at the level of the treatment center during a visit. Participants were administered a structured questionnaire and 3 mL of venous blood was collected from each, into a dry tube for serological diagnosis of *T. gondii* infection, and another 3 mL collected into Ethylenediaminetetraacetic Acid (EDTA) tube for measurement of CD4+ T-cell count.

Administration of questionnaires

Ten questionnaires were pretested at the treatment center of Ndop district hospital in the North West region of Cameroon in June 2018. After the pretest exercise, the questionnaire was adapted

to be completed within 7-10 minutes including keeping the question of age as an open-ended question. Validated questionnaires were administered to participants of all age groups to obtain data on demography (age of participants, gender, marital status, religion, level of education and occupation), risk factors and if participants had any clinical signs and symptoms of *T. gondii* infection, or a history of the infection. Risk factors investigated in this study were; duration on ARV, history of default to HIV/AIDS therapy, availability of cats in participant's home or neighborhood, garden ownership and nature of participant's contact with garden soil, consumption of raw and/or inadequately cooked meat and/or vegetables, source of domestic water supply, and treatment and storage condition of drinking water.

Laboratory analysis

Serological diagnosis of *T. gondii* infection

Serological screening for Toxoplasma infection was achieved using Aria Toxo IgG/IgM Combo Rapid Test with accuracy of 94.9% and 97.8% IgG and I~~GM~~ respectively (CTK Biotech, Inc., San Diego California, USA) according to the manufacturer's instructions [13]. The kit is a lateral flow chromatographic immunoassay; it detects simultaneously the presence of *T. gondii* IgG and IgM specific antibodies in patient's blood. Antibodies against *T. gondii* in patient's serum, binds to recombinant *T. gondii* antigen conjugated with colloidal gold and the coloured complex is captured in the test window coated with mouse anti-human antibody. In essence, 10 uL of serum obtained from patient's blood was applied to the sample pad alongside 70 uL of kit's diluent and the result was read after 10 minutes.

The amount of ***T. gondii*** IgM antibodies was estimated for IgM positive samples using the Biorex diagnostic ***T. gondii*** [illegible] (Antrim, United Kingdom) following manufacturer's instructions. The

Toxoplasma IgM enzyme immunosorbent assay (EIA) Test kit is a solid phase EIA based on immunocapture of IgM antibodies in patient's serum. The presence of *T. gondii* specific antibodies in patient's serum complexes with added recombinant *T. gondii* antigen-enzyme conjugate and reveals a coloured product upon addition of substrate. In brief, following addition of 100 uL of negative control, cut-off calibrator and positive control in appropriate wells, 100 uL of specimen diluent and 5 uL of specimen was added to each of the other wells followed by incubation at 37^0C for 30 minutes and repeated washing. Except for the blank well, 100 uL of conjugate was added to each well and incubated at 37^0C for 30 minutes, followed by repeated washing; after which 50uL of substrate A and 50uL of substrate B were added, and microwell plate was incubated at 37^0C for 10 minutes. The reaction was stopped by addition of 50 uL of stop solution and the optical density was read at 450nm and 650 nm within 30 minutes, using the Emax Precision Microplate ELISA reader (Molecular Divices, California, USA).

The amount of IgG antibodies specific for *T. gondii* in the IgM positive samples was estimated using the Erbalisa® Toxoplasma IgG (Calbiotec, California, USA) according to manufacturer's instructions. The assay is composed of pre-coated wells with purified *T. gondii* antigen, to which patient's serum is added for *T. gondii* specific antibodies to bind, if present and an enzyme conjugate is added which releases a coloured product upon addition of substrate. The assay was carried out by dispensing 100 uL of diluted sample, calibrator, controls and diluent or blank into appropriate wells, and incubated at room temperature for 20 minutes, before washing three times. This was followed by addition of enzyme conjugate and incubation was done at room temperature for 20 minutes before washing. One hundred microlitres of TMB substrate was added to each well and incubated at room temperature for 10 minutes before the reaction was stopped using 100 uL

of stop solution. The optical density was read at 450 nm and 650 nm using Emax Precision Microplate ELISA reader (Molecular Divices, California, USA).

Measurement of CD4+ T-cell count

The Pima™ Analyser was used (SN; PIMA-A-005920; Alere Technology Gmbh Loebstedter Street 103-105 Jena, Germany), in the measurement of CD4+ T-cell count, which employs a static image analysis. In essence plasma obtained from whole blood collected in EDTA tube was kept at 18^0C and analysis was done within 24hrs. Plasma sample was loaded to PIMA CD4+ test cartridge and ran in the analyser following insertion of test cartridge. Results were automatically calculated and absolute T-cell counts displayed.

Ethical considerations

Ethical clearance for this study was obtained from the University of Buea, Faculty of Health Science (FHS) Institutional Review Board (IRB), reference number 2018/2244UB/SG/IRB/FHS and the Bamenda Regional Hospital Institutional Review Board, reference number 26/APP/RDPH/RHB/IRB. Administrative authorization was obtained from the Regional Delegation of Public Health for the North West Region (RDPH), reference number 233/ATT/NWR/RDPH. Only consenting participants from the study population were recruited into the study, and they all signed the informed consent form. For participants below the age of 21 years old, a signed ascent form was also obtained from their parents or guardians to enable them take part in the research. The participant's selection exercise was completely a random process.

Statistical analysis.

The data were analyzed using Microsoft excel 2010 and *IBM SPSS* version 25 with significance level judged at $p<0.05$. Descriptive statistics was computed to establish the frequency of each

response type in the sociodemographic characteristic and the sero-prevalence in the study population. Independent variables (socioeconomic, demographic and nutritional risk factors) were analyzed using bivariate and multivariate logistic regression to eliminate confounders and ascertain the level of significance as suspected risk factors of *T. gondii* infection. A one-way ANOVA test was conducted to compare the difference between the mean CD4+ T cell count groups of HIV/AIDS patients who were either ***T. gondii*** infection negative, chronic toxoplasma infection or acute toxoplasma infection. Linear regression analysis was computed to establish relationships between IgM and IgG antibody levels against *T. gondii* with CD4+ T cell count.

Results

The overall seroprevalence of *T. gondii* in the BHD of the North West Region of Cameroon was 50.5% (*CI*=45.3%-55.7%). The prevalence of chronic (IgG antibody) toxoplasma infection was 45.8% and acute (IgM antibody) infection was 6.5%. The ages of the study participants ranged from 12 to 73 years with an overall mean age of 41.83±13.41years. Majority of the participants, 56.9% (185) were above the age of 41 years, while 43.1% (140) HIV patients were below the age of 41 years. A Pearson Chi-Square test indicated that there was a significant difference in the seroprevalence of *T. gondii* infection across the age groups of HIV patient χ^2 (2) =8.180, $p = 0.017$, with older patients having a higher prevalence (Table 1).

Majority of HIV patients recruited for the study were females 71.1% (231), as compared to males 28.9% (94). One hundred and twenty-seven (55%) females as compared to 43 (45.7%) males were positive for *T. gondii* infection. A Pearson Chi-Square test analysis indicated that there was no statistically significant difference between the number of male and female HIV patients who were positive for *T. gondii* infection χ2 (1) =2.283, $p = 0.131$.

Table 1. Prevalence of *T. gondii* by age, antibodies, and gender in the BHD.

Characteristics	Positive No (%).	Negative No (%)	Total No (%)	χ2	*p-value*
**Antibodies**					
**IgG only**	149(45.8)	176(54.2)	325(100)	-	-
**IgM only**	6(1.8)	319(98.2)	325(100)	-	-
**IgG/IgM**	15(4.6)	310(95.4)	325(100)	-	-
**Gender**					
**Female**	127(55.0)	104(45.0)	231(100)	2.283	0.131
**Male**	43(45.7)	51(54.3)	94(100)	-	-
**Age**					
**≤21**	10 (3.1)	20 (6.2)	30(9.3)	8.180	0.017
**22-40**	52(16)	58 (17.8)	110(33.8)	-	-
**41≥**	108 (33.2)	77 (23.7)	185(56.9)	-	-

%=percentage, χ2=chi square.

Risk factors of *T. gondii* infection in the study population.

In the bivariate and multivariate logistic regression analysis, factors that were statistically significantly associated to the development of ***T. gondii*** in HIV patients included demographic

factors; marital status (*p*=0.0003) and socioeconomic factors; contact with garden soil (*p*=0.0062), and garden ownership (*p*=0.009)

Demographic factors associated with *T. gondii* infection.

T. gondii infection was significantly more prevalent among HIV patients who were Single/Divorced/widow(er) 54.5% (177/325) (Table 2), compared to HIV patients who were married 45.5% (148/325). The risk of developing Toxoplasma infection was about 2.4 times significantly higher among HIV positive patients who were either Single/Divorced/widow(er) (*OR*= 2.41, *CI*=1.49-3.88, *p*=0.0003), compared to patients who were married.

Table 2. Association of *T. gondii* infection and demographic factors among HIV patients in the BHD.

**Variable**	**Seroprevalence**	**Risk factors for *T. gondii* seroprevalence**			
	**No (%)**	**Bivariate Analysis**		**Multivariate Analysis**	
		***COR* (95%CI)**	***p-value***	***AOR* (95%CI)**	***p-value***
**Marital status**					
Married	148 (45.5)	1			
Single/Divorce/ widow(er)	177 (54.5)	1.81 (1.17-2.83)	0.008	2.41(1.49-3.88)	0.0003
**Age**					
**≤21**	30 (9.2)				

**22-40**	110 (33.8)	1.79(0.77-4.18	0.176
**≥41**	185 (57)	2.74(1.22-6.19)	0.015
**Occupation**			
Business	87 (26.8)	1	
Farming	93 (28.6)	1.24(0.69-2.23)	0.479
Others	117 (36)	1.05(0.60-1.83)	0.858
Student	28 (8.6)	0.44(0.18-1.09)	0.075

COR=Crude odd ratio, AOR=Adjusted odd ratio, %= percentage, CI=Confidence Interval.

Nutritional factors associated with *T. gondii* infection.

In the bivariate logistic regression, no nutritional factor (consumption of improperly cooked or raw meat *p*=0.7976 and consumption of improperly cooked vegetable or salad *p*=0.1236) was significantly associated to the development of toxoplasma infection (Table 3).

Table 3: Association of *T. gondii* infection and nutritional factors among HIV patients in the BHD.

Variable	**Seroprevalence**	**Risk factors for *T. gondii* seroprevalence**	
	**No (%)**	**Bivariate Analysis**	
		***COR* (95%CI)**	***p-value***

Consumption of raw/poorly cooked meat			
No idea	85 (26.1)	1	
No	189 (58.2)	1.07 (0.6405-1.7853)	0.7976
Yes	51 (15.7)	0.77 (0.3815-1.5366)	0.4525
Consumption of raw/poorly cooked vegetable			
no	67 (20.6)	1	
Yes	258 (79.4)	1.53 (0.8904-2.6310)	0.1236

COR=Crude odd ratio, %= percentage, CI=Confidence Interval.

Socio-economic factors associated with Toxoplasma infection.

The proportion of HIV patients who presented with Toxoplasma infection (Table 4), and had been in contact with garden soil 74.2% (241/325) was significantly higher compared to HIV patients who presented with Toxoplasma infection without contact with garden soil 25.8% (84/325), (*p*=0.0062). The risk of developing toxoplasmosis was found to be about 7 times significantly higher among HIV patients who had been in contact with garden soil (*OR*=7.03, *CI*=1.74-28.79, *p*=0.0062), compared to HIV patients who had no contact with garden soil.

Majority of Toxoplasma infected HIV patients owned gardens 75.7% (246/325), compared to those who did not own a garden 24.3% (79/325), (*p*=0.009). The risk of developing toxoplasma infection was found to be about 2 times significantly higher among HIV patients who owned gardens (*OR*=2.19, *CI*=1.21-3.96, *p*=0.009), compared to HIV patients who did not own a garden.

Table 4. Association of Toxoplasma infection and socio-economic factors among HIV patients in the BHD.

Variable	Seroprevalence	Risk factors to *T. gondii* seroprevalence			
	No (%)	Bivariate Analysis		Multivariate Analysis	
		COR (95%CI)	*P-value*	AOR (95%CI)	*P-value*
**Contact with garden soil**					
No	84(25.8)	1			
Yes	241(74.2)	3.44(2.02-5.87)	<0.001	7.03(1.74-28.79)	0.0062
**Owns a garden**					
No	79 (24.3)	1			
Yes	246 (75.7)	2.63(1.55-4.46)	0.0003	2.19(1.21-3.96)	0.009
**Owns a cat**					
No	218 (67.1)	1			
Yes	107 (32.9)	1.60(1.00-2.56)	0.049		
**Neighbour owns a cat**					
No	71 (21.2)	1			

Yes	254 (78.2)	2.07(1.20-3.54)	0.008

COR=Crude odd ratio, AOR=Adjusted odd ratio, %= percentage, CI=Confidence Interval.

Determination of CD4+ T-cell count as a predictor of acute Toxoplasma infection.

One-way analysis of variance (ANOVA) test indicated that there was no significant difference ($p = 0.909$) between the mean CD4+ T-cell count of HIV patients who were negative for toxoplasmosis and those who were chronically infected or acutely infected with *T. gondii* infection (Figure 1). Toxoplasma infection was not significantly associated with CD4+ T-cell counts ≤200cells/mL ($p=0.536$).

Fig 1. Mean CD4+ T-cell counts between different status of Toxoplasma infection.

Toxoplasma negative = participants with neither IgM nor IgG antibodies against T. gondii infection.

Chronic Toxoplasma Infection = participants who were positive only for IgG antibodies against T. gondii.

Acute Toxoplasma Infection = participants who were at least positive for IgM antibodies against T. gondii infection

Relationship between IgM antibodies against Toxoplasma infection and CD4+ T-cell count in HIV patients with *T. gondii* infection

Amongst the 21 patients with acute infection (positive for IgM antibodies to *T. gondii*), IgM antibody titers decreased with increasing CD4+ T-cell counts (r=0.193), but there was no statistically significant difference (p=0.401) in IgM antibody titer at different levels of CD4+ T-cell counts (Figure 2).

Fig 2. IgM antibodies against Toxoplasma infection and CD4+ T cell count in HIV patients

Relationship between IgG antibodies against Toxoplasma infection and CD4+ T cell count in HIV patients with acute toxoplasmosis.

Amongst the 21 HIV patients acutely infected with *T. gondii*, there was an increase in IgG antibody titers as the amount of CD4+ T cell counts increased, but there was no linear relationship (p=0.519) as seen in figure 3.

Fig 3. IgG antibodies against Toxoplasma infection and CD4+ T cell count in HIV patients.

Discussion

Results from this study revealed a toxoplasma infection prevalence of 50.5% (164/325). This is less than the prevalence reported in other studies where the prevalence of latent toxoplasmosis was 93.3% in Addis Ababa, Ethiopia, [14]; 60% in Muttu Karl Hospital, Gonder, Ethiopia [15]; 96.3% in North Iran [16] and 69.9% in HIV patients attending the University Teaching Hospital in Yaoundé, Cameroon [11] . The high prevalence of chronic Toxoplasma infection (45.8%) relative to acute infection (6.5%) can be explained by the improved HIV treatment package which reduces mortality and the duration of immune suppression in HIV patients, thus prolonging life expectancy and increasing the number of Toxoplasma chronically infected patients.

Toxoplasma infection was found to be significantly associated (p=0.0062) with HIV patients who had contact with garden soil (74.2%) and those who owned gardens (75.7%, p=0.009). However, the infection was not significantly associated with consumption of raw or inadequately cooked meat (p=0.45), and consumption of raw or inadequately cooked vegetable (p=0.1236). This finding is contrary to what was obtained in Ethiopia amongst a similar group of patients, where anti-*T. gondii* seropositivity was significantly associated with raw meat consumption (p= 0.025) [17].

Consumption of raw meat is not a common practice in the BHD as most of the staple dishes in this locality entail rigorous cooking. In this study; age, cat ownership or neighbors owning cat, were not significantly associated with toxoplasma infection following multivariate analysis. This is contrary to the observation amongst HIV infected women in Ethiopia where the age group 28 to 37 years was a significant risk factor for toxoplasma infection [18]. In the same light, studies carried out in Lagos, Nigeria showed that toxoplasma infection was associated with participants living in close proximity with cats (*p*=0.013), contradicting the findings from this study [19]. This study also showed that Toxoplasma infection was significantly associated with single/divorce/widow(er) (*p*=0.0003), which is contrary to observations in Southern Iran where marital status was not a risk factor for the disease [20]. These findings suggest that the epidemiology of *T. gondii* infection may vary in different settings.

Although other studies [21, 22], have shown that CD4+ T cell count threshold of ≤200cells/mL was indicative of onset of clinical manifestations of toxoplasma infection, in this study, it was observed that there was no significant difference between the mean CD4+ T-cell count of non-infected, chronically infected and acutely infected patients of toxoplasma infection (p = 0.909). The fact that there was no significant correlation between CD4+ T-cell count and IgM antibody titer (r=0.193, p=0.401) suggests that the quantity of IgM antibodies elicited in acutely infected patients is independent of the level of CD4+ T-cells present, although a study in South Brazil reported that CD4+ T-cell count ≤350cells/mm^3 of blood, was significantly associated (p=0.001) with development of acute toxoplasmosis [23]. IgG antibody titer showed a positive correlation with increasing CD4+ T-cells; however, it was not significant (r= 0.149, p=0.519). This suggest that CD4+ T-cell count does not differ from one serological status to the other in *T. gondii*

infection. This suggests that IgG antibody titer cannot be used to predict CD4+ T-cell count in *Toxoplasma gondii* patients presenting with IgG and IgM antibodies.

Conclusion:

The prevalence of *T. gondii* is still high, despite the successes recorded by the package put in place by WHO through the health system to control and manage opportunistic infections among HIV/AIDS patients. These findings therefore have profound clinical and epidemiological significance and call for more urgent action.

Acknowledgement.

We wish to thank the staff of the Bamenda Regional Hospital HIV/AIDS treatment center for their collaboration and the Biotechnology Unit, Faculty of Science, University of Buea for helpful ELIZA laboratory analysis

References

1. Basavaraju, A. Toxoplasmosis in HIV infection: An overview. Tropical Parasitology. 2016, 6, 129-35.
2. Ayoade F. & Joel C. A. S. HIV-1 Associated Opportunistic Infections, Toxoplasmosis. Last updated October 27, 2018. In StatPearls (Internet). *Treasure Island (FL): StatPearls Publishing*; Available from: https://www.ncbi.nlm.nih.gov/books/NBK441877/
3. Galvan-Ramirez, M. L., P., L. R. R., Agraz, S. Y. L., Ávila, L. M. S., R., A. S. A., Corella, D. B., Remirez, B. J. R. & S., R. T. Seroepidemiology of toxoplasmosis in high-school students in the metropolitan area of Guadalajara, Jalisco, Mexico. Scientific Medicine. 2010, **20,** 59-63.

4. Emivi, T. B., Mlatovi, D., Holoud, M., Abiba, K. B., Agnon, B., Kodjo, A., Messanvi, G. Seroprevalence and rick factors of toxoplasmosis in Togo. European Scientific Journal. 2018, (**14**) 1857–7881

5. Fan, C. K., Lee, L. W., Liao, C. W., Huang, Y. C., Lee, Y. L., Chang, Y. T., Ramos José Da Costa, A. D. S., Gil, V., Chi, L. H., Nara, T., Tsubouchi, A. & Akinwale, O. P. *T. gondii* infection: relationship between seroprevalence and risk factors among primary school children in the capital areas of Democratic Republic of São Tomé and Príncipe, West Africa. BMC Parasites & Vectors. 2012; 5, 141.

6. Jeffrey L. Jones, Deanna Kruszon-Moran, Scott Elder, Hilda N. Rivera, Cindy Press, Jose G. Montoya, and Geraldine M. McQuillan. *T. gondii* Infection in the United States, 2011–2014. American Journal Tropical Medicine and Hygiene. 2018; 98(**2**), ~~2018, pp.~~ 551–557

7. Jose Ernesto Vidal. HIV-Related Toxoplasmoisis Revisited: current Concepts and Controversies of Old diseases. Journal of the International Association of Providers of AIDS Care. 2019. 2325-9582.

8. Ayoade, F., Joel C. A. S. HIV-1 Associated Opportunistic Infections toxoplasmosis. Last updated 2020 April 27.

9. Ardeleanu, A., Roşca, O. & Sandu, M. Severe sepsis and cerebral toxoplasmosis in an HIV-infected late presenter patient. BMC Infectious Diseases. 2013; 13(**1**)**,** 8.

10. Robert-Gangneux F & Darde, M, L. Epidemiology of and diagnostic strategies for toxoplasmosis. Clinical Microbiology Reviews 2012; 25: 264–96.

11. Jules, C. N., Anna L. Njunda., Dickson S. Nsagha., Henri L. Kamga., Patrick E. Weledji., Che., V. B. Toxoplasma antibodies amongst HIV/AIDS patients attending the University Teaching Hospital Yaounde in Cameroon. African Journal of Clinical and Experimental Microbiology. 2011; 12(**3**) 1119-123

12. Suresh, K. & Chandrashekara, S. Sample size estimation and power analysis for clinical research studies. Journal of Human Reproductive Sciences. 2012*;* 5, 7-13

13. CTK Biotech, Inc. OnSite™ Toxo IgG/IgM combo Rapid Test-(Serum/Plasma/Whole Blood). 2016. *Last accessed 18 September 2020.*

14. Techalew, S., Mekashaw, T., Endale, T., Belete, T. & Ashenafi, T. Sero-prevalence of latent *T. gondii* infection among HIV-infected and HIV-uninfected people in Addis Ababa, Ethiopia: A comparative cross-sectional study. BioMed Central Research Notes. 2009; 2, 692-700.

15. Yesuf, K. M. & Melese, Z. T. Prevalence of Toxoplasmosis in HIV/AIDS Patients in Mettu Karl Hospital. American Journal of Health Research. 2015; 3(**3),** 182-88.

16. Rahimi, M. T., Mahdavi, S. A., Javadian, B., Rezaei, R., Moosazadeh, M., Khademlou, M., Seyedpour, S. H. & Syadatpanah, A. High Seroprevalence of *T. gondii* Antibody in HIV/AIDS Individuals from North of Iran. Iran Journal of Parasitology. 2015; 10(**8),** 584-589.

17. Dechassa T., Mukarim A., Tadesse M. and Moti Y. Anti-*Toxoplasma* antibodies prevalence and associated risk factors among HIV patients. Asian Pacific Journal of Tropical Medicine. 2019; 9(**5**), 460-464.

18. Zeleke, A. J. and Melsew, Y. A. Seroprevalence of *T. gondii* and associated risk factors among HIV-infected women within reproductive age group at Mizan Aman General Hospital, Southwest Ethiopia: a cross sectional study. Bio Med Central Research Notes. 2017; 10(**1**)70. doi.org/10.1186/s13104-017-2390-6.

19. Jo, Okwuzu & Odunukwe, Nkiruka & Ezechi, Oliver & Gbajabiamila, Titilola & Musa, Adesola & Ezeobi, PM & Oke, BA & Somefun, T & Okoye, RN & Onyeitu, CC & Adetunji, MA & Otubanjo, Olubumi. T. gondii infection in HIV/AIDS: prevalence and risk factors.

African Journal of Clinical and Experimental Microbiology. 2014; 15, 97 10.4314/ajcem.v15i2.7.

20. Rezanezhad H., Sayadi F., Shadmand E., Nasab S. D., Yazdi H. R., Solhjoo K., Kazemi A., Maleki M. and Vasmehjani A. A. Seroprevalence of *T. gondii* among HIV Patients in Jahrom, Southern Iran. Korean Journal of Parasitology. 2017; 55(**1**):99-103. doi: 10.3347/kjp.2017.55.1.99. Epub 2017 Feb 28. PMID: 28285515; PMCID: PMC53652[illegible]

21. Hari, K. R., Modi, M. R., Mochan, A. H. D. & Modi, G. Reduced risk of toxoplasma encephalitis in HIV-infected patients – a prospective study from Gauteng, South Africa. International Journal of STD & AIDS. 2007; 18(**8**)**,** 555-558.

22. Rostami, A., Keshavarz, H., Shojaee, S., Mohebali, M. & Meamar, A. R. (2014). Frequency of *T. gondii* in HIV Positive Patients from West of Iran by ELISA and PCR. Iran Journal of Parasitology. 2014*;* 9**,** 474-81.

23. Silva F. C., Mesenburg M. A., Stauffert D., Bruni M. P. and Farias N. A. R (2017). Risk factors associated with the development of acute *Toxoplasma gondii* infection in positives for HIV/AIDS in South of Brazil: Case-Control study. Brazilian journal of surgery and clinical research. 2017; 20(**2**)12-16.

24. Sayadi, F., Shadmand, E., Nasab, S. D. M., Yazdi, H. R., Solhjoo, K., Kazemi, A., Maleki, M. & Vasmehjani, A. A. Seroprevalence of *T. gondii* among HIV Patients in Jahrom, Southern Iran. Korean Journal of Parasitology. 2017; 55(**1**)**,** 99-103.

List of abbreviation.

AIDS	Acquired Immune Deficiency Syndrome

ANOVA	Analysis of variance
ARV	Anti Retroviral
BHD	Bamenda Health District
CD4+	Cluster of Differentiation
EIA	enzyme immunosorbent assay
EDTA	Ethylenediaminetetraacetic Acid
ELISA	Enzyme Linked Immunosorbent Assay
FHS	Faculty of Health Sciences
HIV	Human Immune Deficiency Virus
IBM	International Business Machine
IgG	Immunoglobulin G
IgM	Immunoglobulin M
IRB	Institutional Review Board
OR	Odd Ratio
RDPH	Regional Delegation of Public Health
T. gondii	*Toxoplasmosis gondii*

WHO	World Health Organization

Figure
Click here to access/download;Figure;Figure 1.docx

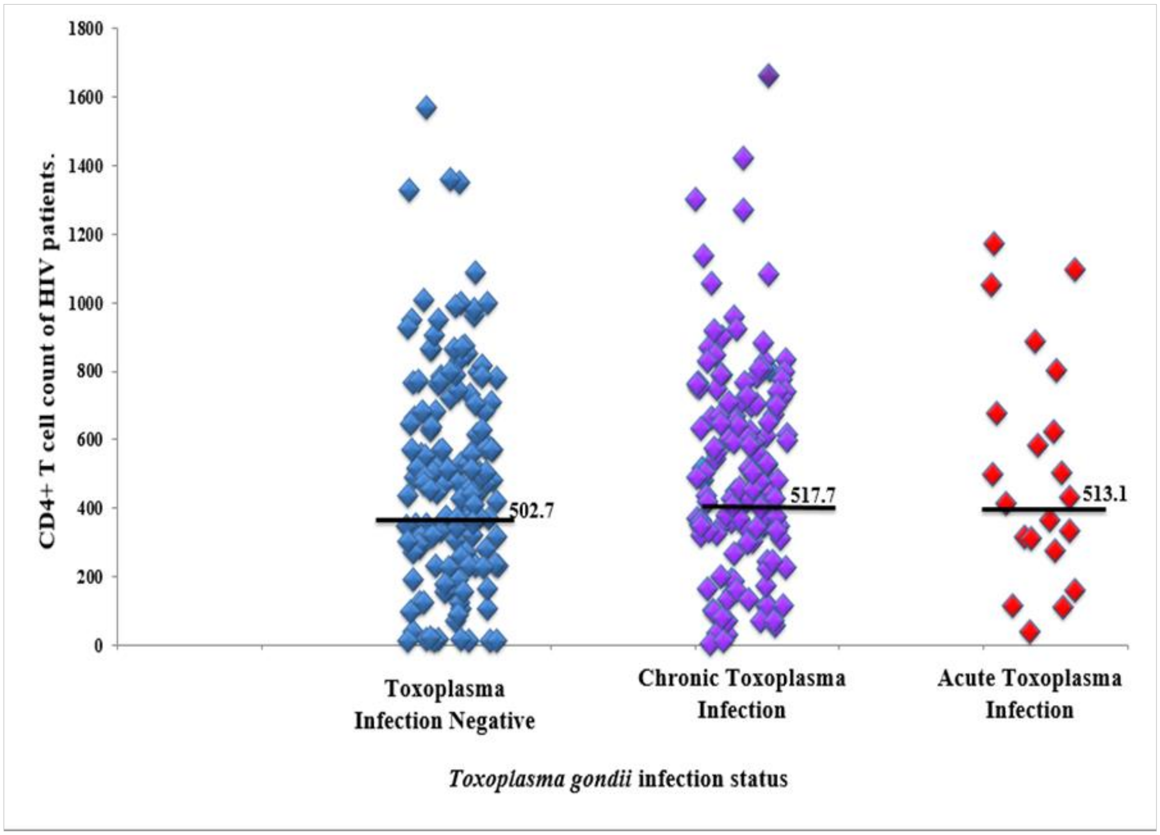

Figure Click here to access/download;Figure;Figure 2.docx

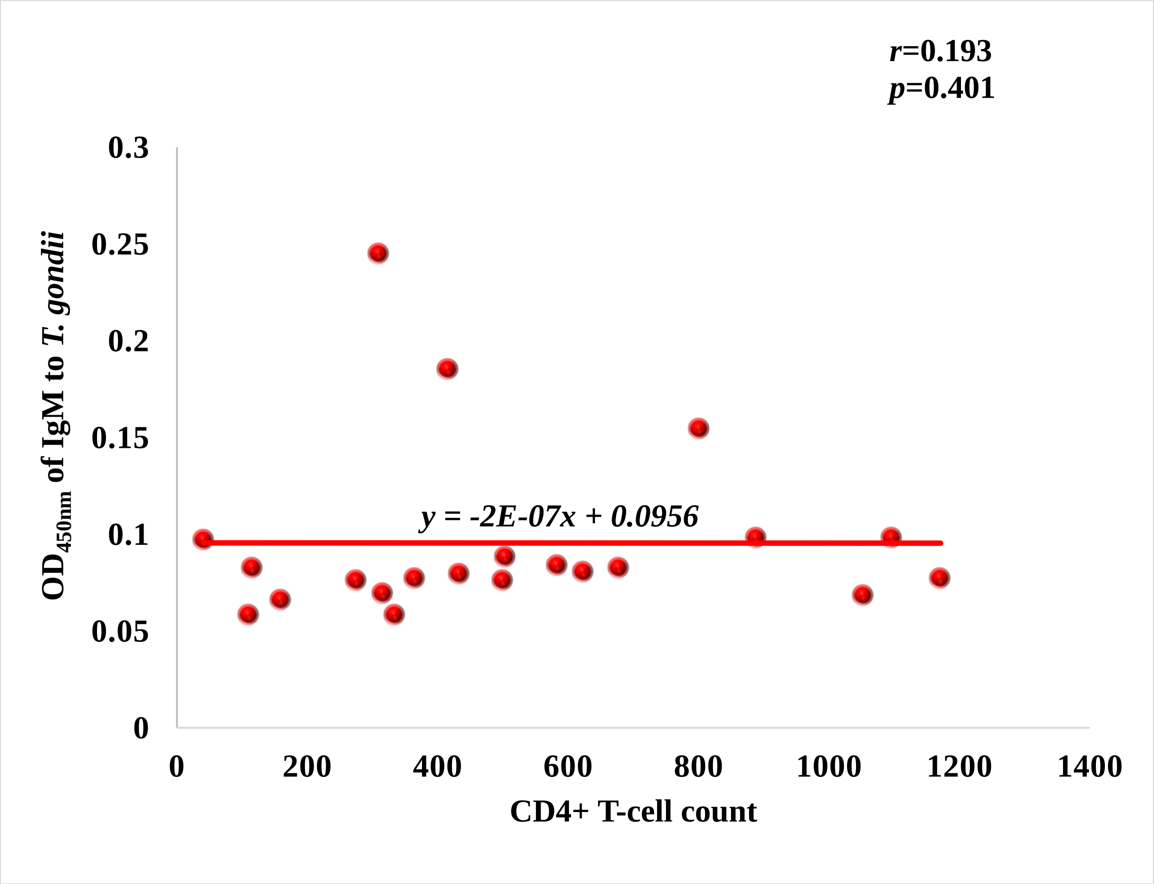

 Click here to access/download;Figure;Figure 3.docx

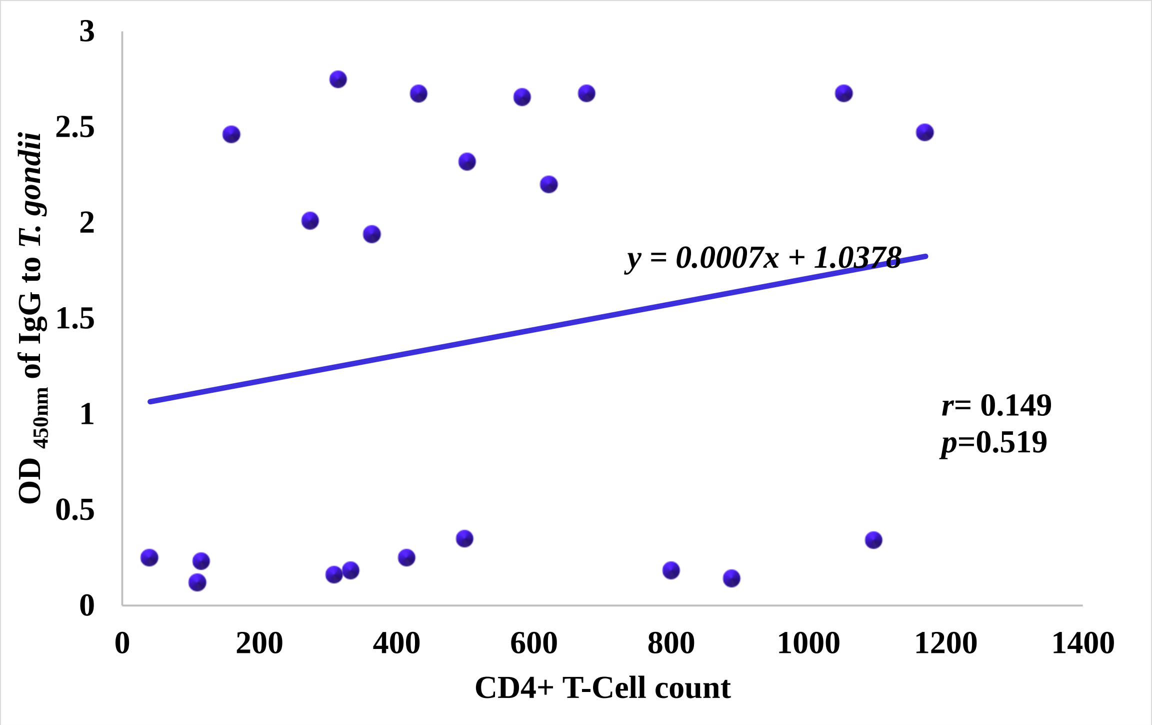

Click here to access/download
Supporting Information - Compressed/ZIP File Archive
Sopporting Information.zip

~~Risk factors for sero-prevalence of Toxoplasma infection and the role of CD4+ T-cell count as a determinant of acute~~ Investigating the risk factors for seroprevalence and the correlation between CD4+ T-cell count and humoral antibody responses to *Toxoplasma gondii* infection amongst HIV patients in the Bamenda Health District, Cameroon.

Eugene Enah Fang[1], Raymond Babila Nyasa [1,2]*, Emmanuel Menang Ndi[2], Denis Zofou[2], Tebit Emmanuel Kwenti[5], Edith Pafoule Lepezeu [4], Vincent P. K. Titanji[2,6] and Roland N. Ndip[1,3]

1. Eugene Enah Fang, M.Sc student in the department of Microbiology and Parasitology, University of Buea, P. O. Box 63, Buea, Cameroon. Email: enah.fang@gmail.com.
2. Raymond Babila Nyasa; Department of Microbiology and Parasitology, University of Buea, P. O. Box 63, Buea, Cameroon. email: nyasab@yahoo.co.uk.
3. Emmanuel Menang Ndi. Biotechnology Unit, Faculty of Science, University of Buea, P.O. Box 63, Buea, South West Region, Cameroon. Email: ndifoinsahlawrence@gmail.com.
4. Denis Zofou. Biotechnology Unit, Faculty of Science, University of Buea, P.O. Box 63, Buea, South West Region, Cameroon. Email: zofden@yahoo.com.
5. Tebit Emmanuel Kwenti. Department of Medical Laboratory Sciences, Faculty of Health Science, University of Buea, P.O. Box 63, Buea, South West Region, Cameroon. Email: kwentitebit@yahoo.com.
6. Pafoule Lepezeu Edith. Bamenda Regional Hospital, P.O. Box 518, Bamenda, North West Region, Cameroon. Email: edithpafoule@gmail.com.
7. Vincent P. K. Titanji. Cameroon Christian University, P.O. Box 5, Bali, North West Region, Cameroon. Email: vpk.titanji@yahoo.com.

8. Roland N. Ndip. Laboratory for Emerging Infectious Diseases, University of Buea, P.O.Box 63, Buea, South West Region, Cameroon. Email: ndip3@yahoo.com; ndip.roland@ubuea.cm.

*Corresponding author: Raymond Babila Nyasa, Department of Microbiology and Parasitology, University of Buea, P. O. Box 63, Buea, South West Region, Cameroon.

Email: nyasab@yahoo.co.uk , Tel. Number: (+237) 677427747

Abstract

Background: Toxoplasmosis is caused by an obligate intracellular tissue protozoan parasite, *Toxoplasma gondii* that infect humans and other warm-blooded animals. Transmission to humans is by eating raw or inadequately cooked infected meat or through ingestion of oocysts that cats have passed in faeces. Studies have shown life-threatening and substantial neurologic damage in immunocompromised patients; however, 80% of humans remain asymptomatic. The aim of this study was to determine the seroprevalence of *T. gondii* infection in HIV positive patients and the risk factors associated with the infection, and to investigate the threshold of CD4+ T-cell count associated with acute *T. gondii* infection in the Bamenda Health District of the North West Region of Cameroon.

Methods: A cross-sectional study was conducted, in which 325 HIV patients were recruited for administration of questionnaire, serological diagnosis of *T. gondii* and measurement of CD4+ T-cell count. Bivariate and multivariate logistic regression was used to identify risk factors associated with *T. gondii* infection while the linear regression was used to investigate the relationship between CD4+ T-cell count and antibody levels against *T. gondii*.

Results: The findings showed that, majority (45.8%) of HIV patients suffered from chronic (IgG antibody) infection, and 6.5% from acute (IgM and IgM/IgG antibody) toxoplasma infection. The overall sero-prevalence of *T. gondii* infection amongst HIV patients was ~~52.3%~~50.5%. On the whole, 43 men (45.7%) and 127 women (55%) presented with anti- *T. gondii* antibodies; however, there was no significant difference amongst males and females who were positive to *T. gondii* infection (*p*=0.131). Marital status (*p*=0.0003), contact with garden soil (*p*=0.0062), and garden ownership (*p*=0.009), were factors that showed significant association with *T. gondii* infection. There was no significant difference (*p* = 0.909) between the mean CD4+ T-cell count of HIV patients negative for toxoplasma infection (502.7 cells/mL), chronically infected with *T. gondii* (517.7 cells/mL) and acutely infected with *T. gondii* (513.1 cells/mL). CD4+ T-cell count was neither a predictor of IgM antibody titer (*r*=0.193, *p*=0.401), nor IgG antibody titer (*r*= 0.149, *p*=0.519) amongst HIV patients acutely infected with *T. gondii*.

Conclusion: The findings from this study underscore the need to implement preventive and control measures to fight against *T. gondii* infection amongst HIV patients in the Bamenda Health District.

Key words: *T. gondii*, sero-prevalence, Bamenda Health District, CD4+ T-cells, HIV.

Introduction.

Toxoplasmosis caused by the obligate intracellular parasite, ~~*T. gondii*~~ *Toxoplasma. gondii* (*T. gondii)* is a globally prevalent zoonotic disease of warm-blooded animals, including man. Within the human population, the infection is clinically silent in immunocompetent host and accounts for focal encephalitis, headache, confusion, motor weakness and fever amongst patients with acquired immunodeficiency syndrome (AIDS) or immuno-compromised patients [1]. In the absence of treatment, the disease progression results in seizures, stupor, coma and finally death [2]. The

infection is contracted through consumption of oocyst in contaminated water, food or from inadequately cooked infected meat. Other means of infection include transfusion and transplantation [3].

~~There is a dearth of information on the sero-prevalence and risk factors associated with the infection amongst human immunodeficiency virus (HIV) patients in the BHD~~ It would be useful to present data regarding the seroprevalence and risk factors associated with the infection amongst human immunodeficiency virus (HIV) of *T. gondii* from different areas of the world. Studies elsewhere have implicated ~~ownership of cat~~ consumption of raw garden produce, level of education and age as risk factors of the infection [4,5.6].

Furthermore, toxoplasmosis has been described as the commonest cerebral opportunistic infection in HIV infected patients [7] and a common cause of mortality amongst severely immune depressed HIV patients [8]. It ~~has been hypothesized that low CD4+ T-cell count~~ is known that a CD4+ T-cell count of ≤200cells/mm^3, accounts for reactivation of latent infection in HIV patients [9]. ~~Because of the opportunistic nature of this infection with obvious sequalae in AIDS patients or immune-compromised patients, it is necessary to determine the relationship between CD4+ T-cell count and antibody titer to current Toxoplasma infection in order to guide management of HIV patients~~. It is therefore rational to think that the magnitude of humoral immune response (IgG and IgM) is a function of CD4+ T cell count, since following *T. gondii* infection, a rise of specific acquired immunity, including humoral immunity accompanied by parasite clearance from the body [10]. If this is true, then high antibody titer in HIV infected toxoplasma patients should positively correlate with CD4+ T cell count, and consequently, either may serve as a marker of the other.

Methods.

Study area and setting

The study was carried out in the Bamenda Health District (BHD), located on the High Western Plateau of Cameroon from August to October 2018. Bamenda is an urban setting, which is both the Administrative Headquarter of Mezam Division and the North West Region of Cameroon. The weather is warm and wet in the rainy season, which last from March to October. In the dry season (November and February), the mornings are generally very cold, and the afternoons are very hot compared to the rainy season. This cosmopolitan city is made up of mainly civil servants and business men and a majority of the population is partly involved in small scale farming. The BHD has a population of over 350,000 inhabitants ~~[10].~~ It comprises of 17 health areas (Figure 1) and 14 public, and 4 faith-based health facilities (1 Baptist, 1 Presbyterian and 2 Catholics) with the Bamenda regional hospital (public) being the leading health facility. Amongst the 18 health facilities, there are only four HIV treatment centers (Bamenda regional hospital, Nkwen sub divisional medicalised health center, Nkwen Baptist hospital and Mezam polyclinic) in BHD.

~~**Fig 1.** ***Adapted from the Bamenda Health District Map published by*** **the Cameroon Ministry of Public Health. Source: National Health Information System Web Portal; URL: .**~~

Study design

A cross sectional study was conducted in the Bamenda health district where data was collected from August to October 2018~~, in the HIV treatment centers of the Bamenda regional hospital and the Nkwen sub divisional medicalised health center, in which participants were recruited by convenient sampling technique.~~ . Participants were recruited by a convenient sampling technique at the HIV treatment centers of the Bamenda regional hospital and the Nkwen sub divisional medicalised health center. ~~The Bamenda Regional Hospital was chosen for this study, because it~~

~~is the reference hospital in the BHD and has the highest number of HIV patients. While the Nkwen sub divisional medicalised health center was randomly selected from the remaining three HIV treatment centers~~ The Bamenda Regional Hospital being the referral hospital and leading in terms of the HIV/AIDS patient population in the health district was purposively chosen for this study, while the Nkwen sub divisional medicalized health center was randomly selected from the other three HIV treatment centers within the health district. Assuming a prevalence of 69.6% from a previous study elsewhere (Yaounde) in Cameroon [11], the sample size was calculated using the formula of Suresh and Chandrashekara [12]. A value of 323 was achieved which was rounded up to 325 during implementation. Three hundred and twenty-five consenting HIV patients 12 years old and above, who were registered at the treatment centers as residents in the BHD were enrolled into the study at the level of the treatment center during ~~a~~ their routine medication appointment visit. Participants were administered a structured questionnaire and 3 mL of venous blood was collected from each, into a dry tube for serological diagnosis of *T. gondii* infection, and another 3 mL collected into Ethylenediaminetetraacetic Acid (EDTA) tube for measurement of CD4+ T-cell count.

Administration of questionnaires

Ten questionnaires were pretested at the treatment center of Ndop district hospital in the North West region of Cameroon in June 2018. After the pretest exercise, the questionnaire was adapted to be completed within 7-10 minutes including keeping the question of age as an open-ended question. ~~Pre-tested questionnaires were administered to participants to obtain data on demography~~ Validated questionnaires were administered to participants of all age groups to obtain data on demography ~~-~~(age of participants, gender, marital status, religion, level of education and occupation), risk factors and if participants had any clinical signs and symptoms of *T. gondii*

infection, or a history of the infection. Risk factors investigated in this study were; duration on ARV, history of default to HIV/AIDS therapy, availability of cats in participant's home or neighborhood, garden ownership and nature of participant's contact with garden soil, consumption of raw and/or inadequately cooked meat and/or vegetables, source of domestic water supply, and treatment and storage condition of drinking water.

Laboratory analysis

Serological diagnosis of *T. gondii* infection

Serological screening for Toxoplasma infection was achieved using Aria Toxo IgG/IgM Combo Rapid Test (CTK Biotech, Inc., San Diego Califonia, USA) according to the manufacturer's instructions [13]. The kit is a lateral flow chromatographic immunoassay; it detects simultaneously the presence of *T. gondii* IgG and IgM specific antibodies in patient's blood. Antibodies against *T. gondii* in patient's serum, binds to recombinant *T. gondii* antigen conjugated with colloidal gold and the coloured complex is captured in the test window coated with mouse anti-human antibody. In essence, 10 uL of serum obtained from patient's blood was applied to the sample pad alongside 70 uL of kit's diluent and the result was read after 10 minutes.

The amount of ~~Toxoplasma~~ ***T. gondii*** IgM antibodies was estimated for IgM positive samples using the Biorex diagnostic ~~Toxo~~ ***T. gondii*** IgM (Antrim, United Kingbom) following manufacturer's instructions. The Toxoplasma IgM enzyme immunosorbent assay (EIA) Test kit is a solid phase EIA based on immunocapture of IgM antibodies in patient's serum. The presence of *T. gondii* specific antibodies in patient's serum complexes with added recombinant *T. gondii* antigen-enzyme conjugate and reveals a coloured product upon addition of substrate. In brief, following addition of 100 uL of negative control, cut-off calibrator and positive control in

appropriate wells, 100 uL of specimen diluent and 5 uL of specimen was added to each of the other wells followed by incubation at 37^0C for 30 minutes and repeated washing. Except for the blank well, 100 uL of conjugate was added to each well and incubated at 37^0C for 30 minutes, followed by repeated washing; after which 50uL of substrate A and 50uL of substrate B were added, and microwell plate incubated at 37^0C for 10 minutes. The reaction was stopped by addition of 50 uL of stop solution and the optical density was read at 450nm and 650 nm within 30 minutes, using the Emax Precision Microplate ELISA reader (Molecular Divices, California, USA).

The amount of IgG antibodies specific for *T. gondii* in the IgM positive samples was estimated using the Erbalisa® Toxoplasma IgG (Calbiotec, California, USA) according to manufacturer's instructions. The assay is composed of pre-coated wells with purified *T. gondii* antigen, to which patient's serum is added for *T. gondii* specific antibodies to bind, if present and an enzyme conjugate is added which releases a coloured product upon addition of substrate. The assay was carried out by dispensing 100 uL of diluted sample, calibrator, controls and diluent or blank into appropriate wells, and incubated at room temperature for 20 minutes, before washing three times. This was followed by addition of enzyme conjugate and incubation was done at room temperature for 20 minutes before washing. One hundred microlitres of TMB substrate was added to each well and incubated at room temperature for 10 minutes before the reaction was stopped using 100 uL of stop solution. The optical density was read at 450 nm and 650 nm using Emax Precision Microplate ELISA reader (Molecular Divices, California, USA).

Measurement of CD4+ T-cell count

The Pima™ Analyser was used (SN; PIMA-A-005920; Alere Technology Gmbh Loebstedter Street 103-105 Jena, Germany), in the measurement of CD4+ T-cell count, which employs a static image analysis. In essence plasma obtained from whole blood collected in EDTA tube was kept at

18^{0}C and analysis was done within 24hrs. Plasma sample was loaded to PIMA CD4+ test cartridge and ran in the analyser following insertion of test cartridge. Results were automatically calculated and absolute T-cell counts displayed.

Ethical considerations

Ethical clearance for this study was obtained from the University of Buea, Faculty of Health Science (FHS) Institutional Review Board (IRB), reference number 2018/2244UB/SG/IRB/FHS and the Bamenda Regional Hospital Institutional Review Board, reference number 26/APP/RDPH/RHB/IRB. Administrative authorization was obtained from the Regional Delegation of Public Health for the North West Region (RDPH), reference number 233/ATT/NWR/RDPH. Only consenting participants from the study population were recruited into the study, and they all signed the informed consent form. For participants below the age of 21 years old, a signed ascent form was also obtained from their parents or guardians to enable them take part in the research. The participant's selection exercise was completely a random process.

Statistical analysis.

The data were analyzed using Microsoft excel 2010 and *IBM SPSS* version 25 with significance level judged at $p<0.05$. Descriptive statistics was computed to establish the frequency of each response type in the sociodemographic characteristic and the sero-prevalence in the study population. Independent variables (socioeconomic, demographic and nutritional risk factors) were analyzed using bivariate and multivariate logistic regression to eliminate confounders and ascertain the level of significance as suspected risk factors of *T. gondii* infection. A one-way ANOVA test was conducted to compare the difference between the mean CD4+ T cell count groups of HIV/AIDS patients who were either ~~toxoplasma~~ ***T. gondii*** infection negative, chronic

toxoplasma infection or acute toxoplasma infection. Linear regression analysis was computed to establish relationships between IgM and IgG antibody levels against *T. gondii* with CD4+ T cell count.

Results

The overall seroprevalence of *T. gondii* in the BHD of the North West Region of Cameroon was ~~52.3% (*CI*=46.88 %-57.68 %).~~ 50.5% (*CI*=45.3%-55.7%). The prevalence of chronic (IgG antibody) toxoplasma infection was 45.8% and acute (IgM antibody) infection was 6.5%. The ages of the study participants ranged from 12 to 73 years with an overall mean age of 41.83±13.41years. Majority of the participants, 56.9% (185) were above the age of 41 years, while 43.1% (140) HIV patients were below the age of 41 years. A Pearson Chi-Square test indicated that there was a significant difference in the seroprevalence of *T. gondii* infection across the age groups of HIV patient χ^2 (2) =8.180, p = 0.017, with older patients having a higher prevalence (Table 1).

Majority of HIV patients recruited for the study were females 71.1% (231), as compared to males 28.9% (94). One hundred and twenty-seven (55%) females as compared to 43 (45.7%) males were positive for *T. gondii* infection. A Pearson Chi-Square test analysis indicated that there was no statistically significant difference between the number of male and female HIV patients who were positive for *T. gondii* infection $\chi 2$ (1) =2.283, p = 0.131.

Table 1. Prevalence of *T. gondii* by age, antibodies, and gender in the BHD.

Characteristics	Positive<br>No (%).	Negative<br>No (%)	Total<br>No (%)	χ2	*p-value*
**Antibodies**					

**IgG only**	149(45.8)	176(54.2)	325(100)	-	-
**IgM only**	6(1.8)	319(98.2)	325(100)	-	-
**IgG/IgM**	15(4.6)	310(95.4)	325(100)	-	-
**Gender**					
**Female**	127(55.0)	104(45.0)	231(100)	2.283	0.131
**Male**	43(45.7)	51(54.3)	94(100)	-	-
**Age**					
**≤21**	10 (3.1)	20 (6.2)	30(9.3)	8.180	0.017
**22-40**	52(16)	58 (17.8)	110(33.8)	-	-
**41≥**	108 (33.2)	77 (23.7)	185(56.9)	-	-

%=percentage, $\chi 2$=chi square.

Risk factors of ~~Toxoplasma~~ *T. gondii* infection in the study population.

In the bivariate and multivariate logistic regression analysis, factors that were statistically significantly associated to the development of ~~Toxoplasmosis~~ ***T. gondii*** in HIV patients included demographic factors; marital status (*p*=0.0003) and socioeconomic factors; contact with garden soil (*p*=0.0062), and garden ownership (*p*=0.009)

Demographic factors associated with ~~Toxoplasma~~ *T. gondii* infection.

~~Toxoplasma~~ ***T. gondii*** infection was significantly more prevalent among HIV patients who were Single/Divorced/widow(er) 54.5% (177/325) (Table 2), compared to HIV patients who were

married 45.5% (148/325). The risk of developing Toxoplasma infection was about 2.4 times significantly higher among HIV positive patients who were either Single/Divorced/widow(er) (*OR*= 2.41, *CI*=1.49-3.88, *p*=0.0003), compared to patients who were married.

Table 2. Association of ~~toxoplasmosis~~ *T. gondii* infection and demographic factors among HIV patients in the BHD.

Variable	Seroprevalence	Risk factors for *T. gondii* seroprevalence			
	No (%)	Bivariate Analysis		Multivariate Analysis	
		COR (95%CI)	*p-value*	*AOR* (95%CI)	*p-value*
**Marital status**					
Married	148 (45.5)	1			
Single/Divorce/ widow(er)	177 (54.5)	1.81 (1.17-2.83)	0.008	2.41(1.49-3.88)	0.0003
**Age**					
**≤21**	30 (9.2)				
**22-40**	110 (33.8)	1.79(0.77-4.18	0.176		
**≥41**	185 (57)	2.74(1.22-6.19)	0.015		
**Occupation**					
Business	87 (26.8)	1			
Farming	93 (28.6)	1.24(0.69-2.23)	0.479		

Others	117 (36)	1.05(0.60-1.83)	0.858
Student	28 (8.6)	0.44(0.18-1.09)	0.075

COR=Crude odd ratio, AOR=Adjusted odd ratio, %= percentage, CI=Confidence Interval.

Nutritional factors associated with ~~Toxoplasma~~ *T. gondii* infection.

In the bivariate logistic regression, no nutritional factor (consumption of improperly cooked or raw meat *p*=0.7976 and consumption of improperly cooked vegetable or salad *p*=0.1236) was significantly associated to the development of toxoplasma infection (Table 3).

Table 3: Association of ~~Toxoplasma~~ *T. gondii* infection and nutritional factors among HIV patients in the BHD.

Variable	**Seroprevalence**	**Risk factors for *T. gondii* seroprevalence**	
	**No (%)**	**Bivariate Analysis**	
		***COR* (95%CI)**	***p-value***
Consumption of raw/poorly cooked meat			
No idea	85 (26.1)	1	
No	189 (58.2)	1.07 (0.6405-1.7853)	0.7976
Yes	51 (15.7)	0.77 (0.3815-1.5366)	0.4525
Consumption of raw/poorly cooked vegetable			

no	67 (20.6)	1	
Yes	258 (79.4)	1.53 (0.8904-2.6310)	0.1236

COR=Crude odd ratio, %= percentage, CI=Confidence Interval.

Socio-economic factors associated with Toxoplasma infection.

The proportion of HIV patients who presented with Toxoplasma infection (Table 4), and had been in contact with garden soil 74.2% (241/325) was significantly higher compared to HIV patients who presented with Toxoplasma infection without contact with garden soil 25.8% (84/325), (*p*=0.0062). The risk of developing toxoplasmosis was found to be about 7 times significantly higher among HIV patients who had been in contact with garden soil (*OR*=7.03, *CI*=1.74-28.79, *p*=0.0062), compared to HIV patients who had no contact with garden soil.

Majority of Toxoplasma infected HIV patients owned gardens 75.7% (246/325), compared to those who did not own a garden 24.3% (79/325), (*p*=0.009). The risk of developing toxoplasmosis was found to be about 2 times significantly higher among HIV patients who owned gardens (*OR*=2.19, *CI*=1.21-3.96, *p*=0.009), compared to HIV patients who did not own a garden.

Table 4. Association of Toxoplasma infection and socio-economic factors among HIV patients in the BHD.

Variable	Seroprevalence	Risk factors to *T. gondii* seroprevalence			
	No (%)	Bivariate Analysis		Multivariate Analysis	
		COR (95%CI)	*P-value*	AOR (95%CI)	*P-value*

**Contact with garden soil**					
No	84(25.8)	1			
Yes	241(74.2)	3.44(2.02-5.87)	<0.001	7.03(1.74-28.79)	0.0062
**Owns a garden**					
No	79 (24.3)	1			
Yes	246 (75.7)	2.63(1.55-4.46)	0.0003	2.19(1.21-3.96)	0.009
**Owns a cat**					
No	218 (67.1)	1			
Yes	107 (32.9)	1.60(1.00-2.56)	0.049		
**Neighbour owns a cat**					
No	71 (21.2)	1			
Yes	254 (78.2)	2.07(1.20-3.54)	0.008		

COR=Crude odd ratio, AOR=Adjusted odd ratio, %= percentage, CI=Confidence Interval.

Determination of CD4+ T-cell count as a predictor of acute Toxoplasma infection.

One-way analysis of variance (ANOVA) test indicated that there was no significant difference (p = 0.909) between the mean CD4+ T-cell count of HIV patients who were negative for

toxoplasmosis and those who were chronically infected or acutely infected with *T. gondii* infection (Figure 2). Toxoplasma infection was not significantly associated with CD4+ T-cell counts ≤200cells/mL (*p=0.536*).

Fig ~~2~~ 1. Mean CD4+ T-cell counts between different status of Toxoplasma infection.

Toxoplasma negative = participants with neither IgM nor IgG antibodies against T. gondii infection.

Chronic Toxoplasma Infection = participants who were positive only for IgG antibodies against T. gondii.

Acute Toxoplasma Infection = participants who were at least positive for IgM antibodies against T. gondii infection

Relationship between IgM antibodies against Toxoplasma infection and CD4+ T-cell count in HIV patients with *T. gondii* infection

Amongst the 21 patients with acute infection (positive for IgM antibodies to *T. gondii*), IgM antibody titers decreased with increasing CD4+ T-cell counts (*r*=0.193), but there was no statistically significant difference (*p*=0.401) in IgM antibody titer at different levels of CD4+ T-cell counts ~~(Figure 3).~~ (Figure 2).

Fig ~~3~~ 2. IgM antibodies against Toxoplasma infection and CD4+ T cell count in HIV patients

Relationship between IgG antibodies against Toxoplasma infection and CD4+ T cell count in HIV patients with acute toxoplasmosis.

Amongst the 21 HIV patients acutely infected with *T. gondii*, there was an increase in IgG antibody titers as the amount of CD4+ T cell counts increased, but there was no linear relationship (*p*=0.519) as seen in ~~figure 4~~ figure 3.

Fig 4. IgG antibodies against Toxoplasma infection and CD4+ T cell count in HIV patients.

Discussion

Results from this study revealed a toxoplasma infection prevalence of ~~52.3% (170/325).~~ 50.5% (164/325). This is less than the prevalence reported in other studies where the prevalence of latent toxoplasmosis was 93.3% in Addis Ababa, Ethiopia, ~~[13]~~ [14]; 60% in Muttu Karl Hospital, Gonder, Ethiopia ~~[14]~~ [15]; 96.3% in North Iran ~~[15]~~[16] and 69.9% in HIV patients attending the University Teaching Hospital in Yaoundé, Cameroon ~~[10]~~ [11]. The high prevalence of chronic Toxoplasma infection (45.8%) relative to acute infection (6.5%) can be explained by the improved HIV treatment package which reduces mortality and the duration of immune suppression in HIV patients, thus prolonging life expectancy and increasing the number of Toxoplasma chronically infected patients.

Toxoplasma infection was found to be significantly associated (*p*=0.0062) with HIV patients who had contact with garden soil (74.2%) and those who owned gardens (75.7%, *p*=0.009). However, the infection was not significantly associated with consumption of raw or inadequately cooked meat (*p*=0.45), and consumption of raw or inadequately cooked vegetable (*p*=0.1236). This finding is contrary to what was obtained in Ethiopia amongst a similar group of patients, where anti-*T. gondii* seropositivity was significantly associated with raw meat consumption (*p*= 0.025) ~~[16]~~[17]. Consumption of raw meat is not a common practice in the BHD as most of the staple dishes in this locality entail rigorous cooking. In this study; age, cat ownership or neighbors

owning cat, were not significantly associated with toxoplasma infection following multivariate analysis. This is contrary to the observation amongst HIV infected women in Ethiopia where the age group 28 to 37 years was a significant risk factor for toxoplasma infection ~~[17].~~ [18] In the same light, studies carried out in Lagos, Nigeria showed that toxoplasma infection was associated with participants living in close proximity with cats (*p*=0.013), contradicting the findings from this study ~~[18].~~ [19]. This study also showed that Toxoplasma infection was significantly associated with single/divorce/widow(er) (*p*=0.0003), which is contrary to observations in Southern Iran where marital status was not a risk factor for the ~~disease~~disease ~~[19]~~[20]. These findings suggest that the epidemiology of *T. gondii* infection may vary in different settings.

Contrary to other studies ~~[20, 21],~~ [21, 22]. which have shown that CD4+ T cell count threshold of ≤200cells/mL was indicative of onset of clinical manifestations of toxoplasma infection, in this study, it was observed that there was no significant difference between the mean CD4+ T-cell count of non-infected, chronically infected and acutely infected patients of toxoplasma infection (p = 0.909). The fact that there was no significant correlation between CD4+ T-cell count and IgM antibody titer (r=0.193, p=0.401) suggests that the quantity of IgM antibodies elicited in acutely infected patients is independent of the level of CD4+ T-cells present, although a study in South Brazil reported that CD4+ T-cell count ≤350cells/mm^3 of blood, was significantly associated (p=0.001) with development of acute toxoplasmosis ~~[22].~~ [23]. IgG antibody titer showed a positive correlation with increasing CD4+ T-cells; however, it was not significant (r= 0.149, p=0.519). This suggests that IgG antibody titer cannot be used to predict progression of the disease amongst acutely infected toxoplasma patients.

Conclusion:

The prevalence of *T. gondii* is still high, despite the successes recorded by the package put in place by WHO through the health system to control and manage opportunistic infections among HIV/AIDS patients. These findings therefore have profound clinical and epidemiological significance and call for more urgent action.

Acknowledgement.

We wish to thank the staff of the Bamenda Regional Hospital HIV/AIDS treatment center for their collaboration and the Biotechnology Unit, Faculty of Science, University of Buea for helpful ELIZA laboratory analysis

References

1. Basavaraju, A. Toxoplasmosis in HIV infection: An overview. Tropical Parasitology. 2016, 6, 129-35.
2. Ayoade F. & Joel C. A. S. HIV-1 Associated Opportunistic Infections, Toxoplasmosis. Last updated October 27, 2018. In StatPearls (Internet). *Treasure Island (FL): StatPearls Publishing*; Available from: https://www.ncbi.nlm.nih.gov/books/NBK441877/
3. Galvan-Ramirez, M. L., P., L. R. R., Agraz, S. Y. L., Ávila, L. M. S., R., A. S. A., Corella, D. B., Remirez, B. J. R. & S., R. T. Seroepidemiology of toxoplasmosis in high-school students in the metropolitan area of Guadalajara, Jalisco, Mexico. Scientific Medicine. 2010, **20,** 59-63.
4. ~~Csep, A., Spineanu, R., Drăghici, S. & Cheregi, S. Relationship between toxoplasmosis in pregnant women and in their feline pets. BMC Infectious Diseases. 2013, 13(**1**), 25~~ Emivi, T. B., Mlatovi, D., Holoud, M., Abiba, K. B., Agnon, B., Kodjo, A., Messanvi, G. Seroprevalence and rick factors of toxoplasmosis in Togo. European Scientific Journal. 2018, (**14**) 1857–7881.
5. Fan, C. K., Lee, L. W., Liao, C. W., Huang, Y. C., Lee, Y. L., Chang, Y. T., Ramos José Da Costa, A. D. S., Gil, V., Chi, L. H., Nara, T., Tsubouchi, A. & Akinwale, O. P. *T. gondii*

infection: relationship between seroprevalence and risk factors among primary schoolchildren in the capital areas of Democratic Republic of São Tomé and Príncipe, West Africa. BMC Veterinary Research. 2012; 5, 141.

6. Jeffrey L. Jones, Deanna Kruszon-Moran, Scott Elder, Hilda N. Rivera, Cindy Press, Jose G. Montoya, and Geraldine M. McQuillan. *T. gondii* Infection in the United States, 2011–2014. American Journal Tropical Medicine and Hygiene. 2018; 98(**2**), 2018, pp. 551–557

7. ~~Ardeleanu, A., Roşca, O. & Sandu, M. Severe sepsis and cerebral toxoplasmosis in an HIV-infected late presenter patient. BMC Infectious Diseases. 2013; 13(**1**), 8~~ Jose Ernesto Vidal. HIV-Related Toxoplasmoisis Revisited: current Concepts and Controversies of Old diseases. Journal of the International Association of Providers of AIDS Care. 2019. 2325-9582.

8. Sayadi, F., Shadmand, E., Nasab, S. D. M., Yazdi, H. R., Solhjoo, K., Kazemi, A., Maleki, M. & Vasmehjani, A. A. Seroprevalence of *T. gondii* among HIV Patients in Jahrom, Southern Iran. Korean Journal of Parasitology. 2017; 55(**1**)**,** 99-103.

9. Saloni Garg, Babita Sharma, Rameshwari Bithu, Nitya Vyas. Seroprevalence of IgM and IgG antibodies to toxoplasma infection in human immunodeficiency virus-positive antiretroviral therapy-naïve individuals. *Muller Journal of medical science and research*; 2019; 10(1)8-12. DOI**:** 10.4103/mjmsr.mjmsr_6_18.

10. ~~Cameroon Ministry of Public Health; Health Map, National Health Information System Web Portal; URL: https://www.dhis-minsante-cm.org/portal/index.html, accessed on February 19th, 2020, 9:50 a.m~~ Robert-Gangneux F & Darde, M, L. Epidemiology of and diagnostic strategies for toxoplasmosis. Clinical Microbiology Reviews 2012; 25: 264–96. .

11. Jules, C. N., Anna L. Njunda., Dickson S. Nsagha., Henri L. Kamga., Patrick E. Weledji., Che., V. B. Toxoplasma antibodies amongst HIV/AIDS patients attending the University Teaching

Hospital Yaounde in Cameroon. African Journal of Clinical and Experimental Microbiology. 2011; 12(**3**) 1119-123

12. Suresh, K. & Chandrashekara, S. Sample size estimation and power analysis for clinical research studies. *Journal of Human Reproductive Sciences. 2012;* 5, 7-13

13. CTK Biotech, Inc. OnSite™ Toxo IgG/IgM combo Rapid Test-(Serum/Plasma/Whole Blood). 2016. *Last accessed 18 September 2020.*

~~13.~~14. Techalew, S., Mekashaw, T., Endale, T., Belete, T. & Ashenafi, T. Sero-prevalence of latent *T. gondii* infection among HIV-infected and HIV-uninfected people in Addis Ababa, Ethiopia: A comparative cross-sectional study. BioMed Central Research Notes. 2009; 2, 692-700.

~~14.~~15. Yesuf, K. M. & Melese, Z. T. Prevalence of Toxoplasmosis in HIV/AIDS Patients in Mettu Karl Hospital. American Journal of Health Research. 2015; 3(**3),** 182-88.

~~15.~~16. Rahimi, M. T., Mahdavi, S. A., Javadian, B., Rezaei, R., Moosazadeh, M., Khademlou, M., Seyedpour, S. H. & Syadatpanah, A. High Seroprevalence of *T. gondii* Antibody in HIV/AIDS Individuals from North of Iran. Iran Journal of Parasitology. 2015; 10(**8),** 584-589.

~~16.~~17. Dechassa T., Mukarim A., Tadesse M. and Moti Y. Anti-*Toxoplasma* antibodies prevalence and associated risk factors among HIV patients. Asian Pacific Journal of Tropical Medicine. 2019; 9(**5**), 460-464.

~~17.~~18. Zeleke, A. J. and Melsew, Y. A. Seroprevalence of *T. gondii* and associated risk factors among HIV-infected women within reproductive age group at Mizan Aman General Hospital, Southwest Ethiopia: a cross sectional study. Bio Med Central Research Notes. 2017; 10(**1**)70. doi.org/10.1186/s13104-017-2390-6.

~~18.~~19. Jo, Okwuzu & Odunukwe, Nkiruka & Ezechi, Oliver & Gbajabiamila, Titilola & Musa, Adesola & Ezeobi, PM & Oke, BA & Somefun, T & Okoye, RN & Onyeitu, CC & Adetunji, MA & Otubanjo, Olubumi. T. gondii infection in HIV/AIDS: prevalence and risk factors. African Journal of Clinical and Experimental Microbiology. 2014; 15. 97. 10.4314/ajcem. v15i2.7.

~~19.~~20. Rezanezhad H., Sayadi F., Shadmand E., Nasab S. D., Yazdi H. R., Solhjoo K., Kazemi A., Maleki M. and Vasmehjani A. A. Seroprevalence of *T. gondii* among HIV Patients in Jahrom, Southern Iran. Korean Journal of Parasitology. 2017; 55(**1**):99-103. doi: 10.3347/kjp.2017.55.1.99. Epub 2017 Feb 28. PMID: 28285515; PMCID: PMC5365268

~~20.~~21. Hari, K. R., Modi, M. R., Mochan, A. H. D. & Modi, G. Reduced risk of toxoplasma encephalitis in HIV-infected patients – a prospective study from Gauteng, South Africa. International Journal of STD & AIDS. 2007; 18(**8**)**,** 555-558.

~~21.~~22. Rostami, A., Keshavarz, H., Shojaee, S., Mohebali, M. & Meamar, A. R. (2014). Frequency of *T. gondii* in HIV Positive Patients from West of Iran by ELISA and PCR. Iran Journal of Parasitology. 2014*;* 9**,** 474-81.

~~22.~~23. Silva F. C., Mesenburg M. A., Stauffert D., Bruni M. P. and Farias N. A. R (2017). Risk factors associated with the development of acute *Toxoplasmosis gondii* infection in positives for HIV/AIDS in South of Brazil: Case-Control study. Brazilian journal of surgery and clinical research. 2017; 20(**2**)12-16

List of abbreviation.

AIDS	Acquired Immune Deficiency Syndrome

ANOVA	Analysis of variance
ARV	~~Anti Retroviral~~Anti-Retroviral
BHD	Bamenda Health District
CD4+	Cluster of Differentiation
EIA	enzyme immunosorbent assay
EDTA	Ethylenediaminetetraacetic Acid
ELISA	Enzyme Linked Immunosorbent Assay
FHS	Faculty of Health Sciences
HIV	Human Immune Deficiency Virus
IBM	International Business Machine
IgG	Immunoglobulin G
IgM	Immunoglobulin M
IRB	Institutional Review Board
OR	Odd Ratio
RDPH	Regional Delegation of Public Health
T. gondii	*Toxoplasmosis gondii*

WHO	World Health Organization

Response to Reviewers.

Dear reviewers kindly fine in this later the response to the questions posted.

The topic of the manuscripts has been modified to "Investigating the risk factors for seroprevalence and the correlation between CD4+ T-cell count and humoral antibody responses to *Toxoplasma gondii* infection amongst HIV patients in the Bamenda Health District, Cameroon" *to* tie the research with the scientific outcome. Information about the pretest procedure of the question is as described in line 124 in the questionnaire administration sub heading.

During analysis, the numeric variables followed an abnormal distribution and the analysis of variance test was used.

Determining the relationship between CD4+ T-cell count to antibody titer was to evaluate the effect of the parasite on the immune system in the study population. The investigator captured the aspect of risk factors to ascertain the epidemiology situation of the parasite in the study population. The investigator used a highly specific and sensitive screening test to ascertain the status of infection of the parasite among the study population. It is also true that, the presence of IgM antibodies is the indication of onset of infection and/or recontamination. The inclusion criteria have been addressed in the revised manuscript line 119 indicating the patients were for their routine drug pick up.

The analysis of CD4+T-cell counts among toxoplasma negative and positive patients was to proof the hypothesis that say immune depression (Low CD4+ T-cell count) futher puts the patients in danger to Toxoplasmosis complications.
