# Supplementary material for: Investigating the risk factors for seroprevalence and the correlation between CD4+ T-cell count and humoral antibody responses to Toxoplasma gondii infection amongst HIV patients in the Bamenda Health District, Cameroon
Source: PLoS One. 2021 Dec 2;16(12):e0256947. doi: 10.1371/journal.pone.0256947 (PMC8638881; doi:10.1371/journal.pone.0256947)
Supplement: Attachment — Submitted filename: Response to Reviewers.docx [file pone.0256947.s002.docx]

Response to Reviewers.

Dear reviewers kindly fine in this later the response to the questions posted.

The topic of the manuscripts has been modified to “Investigating the risk factors for seroprevalence and the correlation between CD4+ T-cell count and humoral antibody responses to *Toxoplasma gondii* infection amongst HIV patients in the Bamenda Health District, Cameroon” *to* tie the research with the scientific outcome. Information about the pretest procedure of the question is as described in line 124 in the questionnaire administration sub heading.

During analysis, the numeric variables followed an abnormal distribution and the analysis of variance test was used.

Determining the relationship between CD4+ T-cell count to antibody titer was to evaluate the effect of the parasite on the immune system in the study population. The investigator captured the aspect of risk factors to ascertain the epidemiology situation of the parasite in the study population. The investigator used a highly specific and sensitive screening test to ascertain the status of infection of the parasite among the study population. It is also true that, the presence of IgM antibodies is the indication of onset of infection and/or recontamination. The inclusion criteria have been addressed in the revised manuscript line 119 indicating the patients were for their routine drug pick up.

The analysis of CD4+T-cell counts among toxoplasma negative and positive patients was to proof the hypothesis that say immune depression (Low CD4+ T-cell count) futher puts the patients in danger to Toxoplasmosis complications.
