# Supplementary material for: Investigating the risk factors for seroprevalence and the correlation between CD4+ T-cell count and humoral antibody responses to Toxoplasma gondii infection amongst HIV patients in the Bamenda Health District, Cameroon
Source: PLoS One. 2021 Dec 2;16(12):e0256947. doi: 10.1371/journal.pone.0256947 (PMC8638881; doi:10.1371/journal.pone.0256947)
Supplement: Attachment — Submitted filename: Response to reviewers.docx [file pone.0256947.s004.docx]

Response to Reviewers.

Dear reviewers’ kindly fine in this later the response to the questions posted.

The average annual temperature of the study area is 19.3^0^c. During the risk factor analysis of the data, the demographic, socioeconomic and nutritional characteristics were all included together in the multivariate analysis. It was done after a bivariate analysis were performed.

The investigator captured the aspect of clinical manifestation by using a questionnaire tool. These questions were asked to the participants to consult them of the history of clinical manifestation event related to that of the illness.
