# Supplementary material for: Investigating the risk factors for seroprevalence and the correlation between CD4+ T-cell count and humoral antibody responses to Toxoplasma gondii infection amongst HIV patients in the Bamenda Health District, Cameroon
Source: PLoS One. 2021 Dec 2;16(12):e0256947. doi: 10.1371/journal.pone.0256947 (PMC8638881; doi:10.1371/journal.pone.0256947)
Supplement: Attachment — Submitted filename: Response to Reviewers.doc [file pone.0256947.s006.doc]

**Responses to Review Comments for Manuscript: PONE-D-20-04369R2**

Dear Sir/Madam,

Thank you for pointing out the corrections that should be done on our manuscript. We have dealt with them as recommended/advised; all the points raised have been addressed; grammatical and typographical errors inclusive. We have equally removed reference 24; this is because it has not been cited anywhere in the manuscript. We have also highlighted the changes introduced. We hope we have answered all the questions and concerns satisfactorily and therefore look forward to your anticipated action.

**REVIEWER 3**

**Comment:** Please delete ‘’ It would be useful to present data regarding the seroprevalence and risk factors associated with the infection amongst human immunodeficiency virus (HIV) of *T. gondii* from different areas of the world.

**Response**: The statement has been deleted and replaced with‘’Knowledge of *T. gondii* seroprevalence and its associated risk factors is important to predict the risk of infection in humans’’ as suggested. Please, see lines 71 – 72 on the revised manuscript with tract changes

Raymond B. NYASSA, PhD

Corresponding author
